# Chinese Version of the Career Adapt-Abilities Scale for Clinical Nurses: A Translation and Psychometric Validation Study

**DOI:** 10.1155/jonm/8198854

**Published:** 2024-11-27

**Authors:** Hui Yu Liang, Hung Da Dai, Jin Yun Chuang, Tzu Yi Tseng, Shu Yu

**Affiliations:** ^1^School of Nursing, National Taipei University of Nursing and Health Sciences, Taipei, Taiwan; ^2^Department of Nursing, Taipei Veterans General Hospital, Taipei, Taiwan; ^3^College of Nursing, National Yang-Ming Chiao Tung University, Taipei, Taiwan; ^4^Department of Nursing, China Medical University Hsinchu Hospital, Hsinchu, Taiwan

**Keywords:** adaptability, career, development, nurses, psychometrics

## Abstract

**Background:** The global environment is continually changing; therefore, adaptability has become a crucial skill in most careers, including nursing. Career adaptability, which is essential to nurses' career development, influences nurse retention. However, to the best of our knowledge, no suitable tool has been developed for assessing the career adaptability of clinical nurses in Taiwan.

**Aim:** To translate the Career Adapt-Abilities Scale (CAAS) into traditional Chinese and validate the psychometric properties of this Chinese version of the CAAS (named CAAS-C).

**Methods:** A two-phase cross-sectional study was conducted. Purposive sampling was used to recruit 584 registered nurses from two teaching hospitals in Taiwan. The CAAS was translated in accordance with a modified version of Brislin's guidelines, that is, through forward translation, back-translation, and expert committee review. Test–retest reliability, internal consistency, content validity, and construct validity were evaluated to assess the psychometric properties of the CAAS-C.

**Results:** The results revealed a content validity index value of 0.96. Confirmatory factor analyses revealed acceptable model fit. The test–retest reliability was excellent (intraclass correlation coefficient = 0.82), and the internal consistency of the CAAS-C was satisfactory (Cronbach's alpha = 0.90–0.96).

**Conclusions:** The CAAS-C is a brief, valid, and reliable instrument for measuring the career adaptability of clinical nurses.

**Implications for Nursing Management:** The CAAS-C can be used to evaluate Taiwanese nurses' career adaptability and develop effective strategies for improving nurses' responsiveness to their rapidly changing work environments, which can improve adaptation and retention.

## 1. Introduction

The global workforce faces an occupational environment that is persistently changing [1]; consequently, employees must continually adapt to changes and challenges in their careers. Individuals who can effectively adapt to changing conditions are more likely to be stably committed to their job and have a successful career [2]. Career adaptability, which has become a key topic of career-related research [3], refers to an individual's ability to adapt to changes and their preparedness to meet their current and future occupational demands [4, 5]. Career adaptability can help individuals cope with the changes and developments in their career. It can also increase their life satisfaction [3], career satisfaction, and career success as well as prevent employee turnover in organizations [6].

Nurses comprise the largest professional group in the health-care sector [7]. Because numerous countries are facing the problem of population aging, registered nurses are in high demand [8]. However, the coronavirus pandemic led to a considerable increase in turnover and intention to leave the profession among nurses due to its effects on their career, and the pandemic has consequently had an adverse effect on the nursing labor market [9, 10]. The International Council of Nurses reported that 20% of the National Nursing Associations affiliated with the council reported that in 2020, one third of nurses were considering leaving the profession within 1 year because of the pandemic [8]. In the Taiwanese registered nurse workforce, turnover and vacancy rates are extremely high compared with what they were prior to the pandemic. Although approximately 5000 newly licensed nursing school graduates enter the nursing workforce annually [11, 12], the increasing turnover rate among registered nurses has resulted in a higher vacancy rate; the vacancy rate in Taiwan (Republic of China, ROC) increased from 4.7% in 2019 to 6.53% in 2022 [13]. This increase in the vacancy rate is higher than those in Western counties [14]. Taiwan (ROC) is also facing the problem of a severe shortage of nurses, which has resulted in temporary elimination of services, admission restrictions, and bed closure in many hospitals [15]. High nurse turnover rates adversely affect the quality of health-care services and the career development and satisfaction of nurses [16–18].

Although nurses are in high demand in Taiwan (ROC), a insufficient number of nurses choose to remain in the workforce. To mitigate this problem, relevant authorities must identify a means of ensuring that Taiwanese nurses are able to adjust and be responsive to dramatic changes in their work environment and develop the skills they require to adapt to complex situations and manage various tasks and environments [19]. Doing so can enable them to provide safe and effective health-care services [16]. Nurses with a higher level of adaptability are generally better equipped to adjust to rapid organizational changes and develop core competencies; they are also more likely to be committed to their career, experience success, and feel satisfaction and job embeddedness [20], which are key factors influencing nurse retention in the workplace [21–26]. Therefore, being able to accurately assess nurses' career adaptability is crucial to improving the retention of nurses, particularly in the context of the aftereffects of the coronavirus pandemic.

To the best of our knowledge, no effective and culturally appropriate tool has yet been developed for assessing the career adaptability of Chinese-speaking clinical nurses in Taiwan (ROC). Although one study translated a Chinese version of the Career Adapt-Abilities Scale (CAAS) to assess the career adaptability of Chinese employees [27], the translated scale was a short-form version comprising only 12 items and was specific to workers who were university graduates in China. Furthermore, the scale was written using simplified Chinese, which is the standard written form of Chinese in China; in Taiwan, traditional Chinese is the standard. Additionally, China and Taiwan have clear differences in terms of their language use and culture, and these differences influence their form of expression in Chinese. Therefore, the aforementioned version of the CAAS may not be applicable to Taiwanese clinical nurses [28]. Therefore, we translated the CAAS into traditional Chinese and evaluated the psychometric properties of this traditional Chinese version of the CAAS (named the CAAS-C) in clinical nurses. The CAAS-C can be used to evaluate and understand the career adaptability of Chinese-speaking nurses in Taiwan. The valid and reliable CAAS-C can enable nurse managers and health-care administrators to precisely assess nurses' career adaptability, which can help them to improve nursing resources and develop effective nurse retention and development strategies.

## 2. Background

Over a decade ago, Savickas [4] proposed the concept of career adaptability as a central construct in career development. This concept subsequently served as the basis of the life‐span, life‐space career theory proposed in one study [4] and Savickas' career construction theory [2]. The term adaptability refers to an individual's capacity and willingness to change to adjust to new or altered circumstances [4], and adaptability is considered to be a critical psychosocial resource [29]. Career adaptability involves personal resources that can assist an individual in adjusting to their current circumstances and that can enable the individual to utilize their occupational personality to adjust to changes in the sequence of their job [4]. Employees' psychosocial resources and transactional competencies influence their adaptive behaviors in various environments [29].

Individuals with high career adaptability are able to successfully navigate unfamiliar and complex environments [4, 29]. Career adaptability can be cultivated through interactions with individuals and the environment [3]. Several studies have indicated that successful development of career adaptability can improve individual and organizational career outcomes [6]. A systematic review reported that successful development of career adaptability resources can lead to more career exploration, higher vocational commitment, and higher career satisfaction and can reduce turnover and employees' intention to leave [6]. Haibo, et al. [30] investigated the associations among career adaptability, organizational success (turnover intention and job performance), and individual career success (career satisfaction and yearly income). Their results revealed that career adaptability positively predicted employees' job performance and subjective and objective career success and negatively predicted turnover intention. Orie and Semeijn [31] explored the associations among career adaptability, organizational embeddedness, and turnover intention, and their results revealed that career adaptability predicted turnover intention and that career adaptability and organizational embeddedness had a significant interacting influence on turnover intention.

Nursing is a field that requires a high level of professional competence; nurses must engage in complex person–environment interactions and manage their professional career [32, 33]. Nurses play a crucial role in ensuring that hospitals provide effective, quality care, and a safe care environment for patients [34]; thus, ensuring the nurse workforce of a hospital is sufficient is essential to the hospital being able to deliver quality care. Research indicates that approximately 40% of nurses struggle to adjust to dynamic nursing environments [35], and lacking the skills and competencies required to respond to change and adapt to different situations is likely to lead to higher turnover intention among nurses [36]. Assessing nurses' career adaptability is crucial to enabling identification of their skills and competencies that must be strengthened and development of interventions that can improve nurses' abilities to adapt.

Psychometric tools can be used to measure nurses' career adaptability. The CAAS, first developed by Savickas and Porfeli [29], is a theory-based tool that can be used to effectively measure career adaptability. The attributes that contribute to career adaptability include career concern, career control, career curiosity, and career confidence [2]. The term career concern refers to an individual's concerns regarding the future of their career. Career control involves an individual's autonomy in determining the direction of their future career. Career curiosity refers to an individual demonstrating initiative and exploring possible future careers. Career confidence involves an individual's belief in their ability to realize their aspirations and achieve their career goals [4, 29]. The original CAAS comprised 44 items; however, to extend the nomological network of career adaptability and facilitate its adaptation for international career development research, Savickas and Porfeli [29] shortened the CAAS to 24 items (International Version 2.0).

The CAAS is a multidimensional, self-administered questionnaire; it is the first tool developed for assessing career adaptability. The CAAS is easy to implement, and its results are easy to interpret [29]. Since the development of the CAAS, several other tools for measuring career adaptability have been introduced. Such tools have been translated and validated for use in numerous countries and regions, including Europe, Switzerland, South Africa, the United States, Germany, Belgium, South Korea, Taiwan, France [29], and Thailand [37]. However, these tools have mainly been employed in studies involving college students, civil servants, and company employees [29, 38]; they have not been applied in those involving nurses. Research indicates that psychometric tools specific to workers in certain fields must be developed and validated [38]; to date, no traditional Chinese version of a psychometric tool has been developed or validated for assessing the career adaptability of clinical nurses in Taiwan. A valid and reliable traditional Chinese-language version of such a tool is required to ensure that nursing managers and health-care administrators can accurately assess nurses' career adaptability. Effectively assessing nurses' adaptive behavior and capacity can enable relevant authorities to strategically strengthen nurses' adaptive capacity and reduce turnover in their organization.

## 3. Methods

### 3.1. The Study

**Figure 1:**
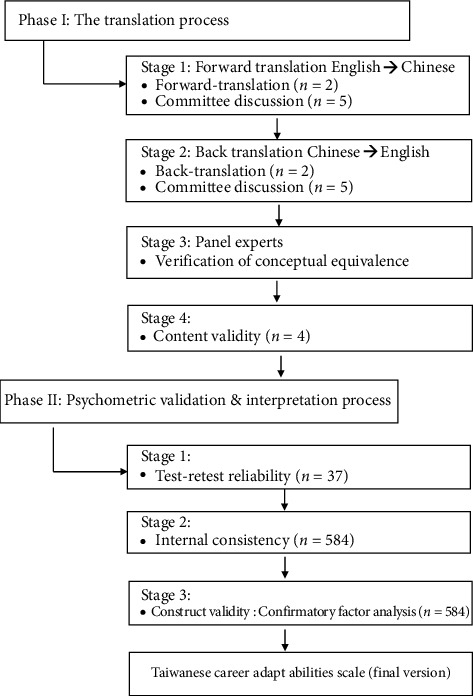
Process followed for translating and validating the Chinese version of the Career Adapt-Abilities Scale for clinical nurses.

In this study, permission was obtained from the author of the original scale for translation [29]. Subsequently, a cross-sectional descriptive study was conducted to assess the measurement properties of the CAAS-C. The study involved two phases: translation and testing of the psychometric validation of the CAAS into Chinese. The validity of the CAAS-C in Taiwan was confirmed (Figure 1).

### 3.2. Phase 1: Translation

The 24-item CAAS (International Version 2.0) was originally developed by Savickas and Porfeli [29] and measures an employee's abilities related to career adaptation in four dimensions, namely, career concern, control, curiosity, and confidence. Each dimension is evaluated using six items, and responses are rated on a five-point Likert scale, with endpoints ranging from 1 *(not strong)* to 5 *(very strong)*. A higher score indicates a greater ability to adapt in one's career. The CAAS has been demonstrated to be psychometrically robust in 13 countries [29] and to have an overall Cronbach's alpha of 0.92 and acceptable goodness of fit (comparative fit index [CFI] = 0.86–0.94, root mean square error of approximation [RMSEA] = 0.046–0.78, and standardized root mean square residual [SRMR] = 0.037–0.070) [29]. The scale is a valid and reliable instrument; therefore, a Chinese translation of the scale would be suitable for use among clinical nurses in Taiwan.

The translation process used in this study was adapted from the methods proposed by Brislin [39] and Jones, et al. [40]. It involved forward translation, back translation, expert committee review, and content validity assessment. First, the original English version of the CAAS was independently translated into Chinese by two bilingual translators. One translator, born in the United Kingdom, had lived in Taiwan for 22 years, held a master's degree, and had worked as a professional English translator, editor, and writer for 19 years. The other translator was an assistant professor with a PhD in nursing, whose first language was Mandarin Chinese.

The two translated versions of the scale were compared and consolidated by a panel of five experts. Feedback from the panel discussion regarding each item assisted the translators in reaching a consensus regarding how the items should be phrased to ensure the highest possible level of accuracy and understandability. The resultant preliminary version of the CAAS-C was independently back-translated by two bilingual translators whose native language was English and who had a cross-language expertise. These translators compared the Chinese and English versions of the scale to assess their conceptual equivalence. The back-translated version was compared with the original English version to identify possible conceptual inconsistencies. Subsequently, the Chinese version was reviewed by an expert committee to ensure that the forward- and back-translated scales were equivalent, and the translated scale was finalized.

Finally, the content validity of the scale was determined through expert scoring [41]. A panel of four experts—a professor of human resource management, an associate professor of career management and counseling, an associate professor of employment and vocational training, and a professor of nursing—evaluated the content validity of the CAAS-C. The clarity and relevance of each item were measured on a four-point Likert scale, with endpoints ranging from 1 *(irrelevant or very unclear)* to 4 *(very relevant or very clear)*.

### 3.3. Phase 2: Psychometric Validation

A cross-sectional descriptive study design was used to validate the psychometric properties of the CAAS-C (Phase 2), which was completed in three steps. In the first step, a test–retest evaluation was performed to determine the stability of the scores for each construct. The sample size for test–retest analysis was determined by the rules [42], which indicated that a minimum of 30 participants was required to calculate the intraclass correlation coefficient (ICC). Considering a drop-out rate of 20%–30% [43], we initially recruited a total sample size of 40 nurses. Ultimately, 37 nurses participated in the test–retest reliability evaluation, with the retest conducted after a 2-week interval.

Subsequently, Cronbach's alpha was calculated to assess the internal consistency of the scales and subscales. In the final step of the validation process, confirmatory factor analysis (CFA) was conducted to examine the relationships between the observed and latent variables in the measurement model [44], in order to evaluate construct validity. Participants were recruited through purposive sampling from two teaching hospitals in northern Taiwan, each with over 400 beds. The number of nurses recruited from each department was based on the number of nurses working in that department. Full-time nurses (aged 20 years and older) working in nursing departments were included in the study, while those from nonnursing departments were excluded due to their generally limited experience in providing direct clinical care to patients. We determined that the sample size required to ensure factor analysis with high loadings per factor and to thereby achieve the goals of this study was 200–400 participants [45]. According to a relevant study, response rates for studies such as this generally range from 50% to 80% [46]. Therefore, we initially invited 662 nurses to participate in this study; 584 were ultimately enrolled, resulting in a response rate of 88.2%.

### 3.4. Data Collection

Data were collected using a self-administered questionnaire. A survey was conducted among Taiwanese clinical nurses between August 1, 2019, and July 31, 2020. The research team explained the purpose of the study to eligible nurses prior to their enrollment at each hospital. The research team distributed printed copies of the questionnaire in sealed envelopes to nurses who agreed to participate in each department of each hospital. To ensure the accuracy and correctness of responses, we established a specific date on which the completed questionnaires were to be returned to the research team.

### 3.5. Statistical Analysis

Data were analyzed using SPSS (version 24.0) and AMOS (version 22.0) for Window (Chicago, IL, USA) software to further validate the scale. The participants' demographic data were presented as frequencies, percentages, means, and standard deviations. Content validity was used to assess whether the items in the scale adequately reflected the concepts being explored, and it was evaluated using content validity index (CVI). Content validity was determined by calculating CVI at both the item level (I-CVI; validity ≥ 0.78) and the scale level (S-CVI; validity ≥ 0.9) [47].

The construct validity of the CAAS-C for clinical nurses was assessed using CFA to validate the model fit between the observed and latent variables. Maximum likelihood estimation was employed to assess the adequacy of the measurement model. A squared standardized factor loading value greater than 0.5 was used to determine the appropriateness of the constructs [48]. Modification indices (MIs) were used to assess the model modifications and acceptability [49]. Model fit was assessed according to the fit indices and acceptable thresholds proposed by Hu and Bentler [50], using the following criteria: *χ*^2^ (with associated degrees of freedom [*df*] and *p* values), relative *χ*^2^ (*χ*^2^/*df*) < 5.0, RMSEA < 0.06, goodness-of-fit index (GFI) > 0.90, adjusted GFI (AGFI) > 0.90, CFI > 0.90, and SRMR < 0.05. To assess the stability of the CAAS-C, test–retest reliability was evaluated using a two-tailed Student's *t*-test. ICCs were calculated to assess the 2-week test–retest reliability, with an alpha value of 0.70 indicating adequate reliability [51]. The internal consistency reliability of the CAAS-C was evaluated using Cronbach's alpha coefficients, with a minimum acceptable alpha value set at 0.70 [45]. For all analyses, the level of statistical significance in this study was set at *p* < 0.05.

### 3.6. Ethical Consideration

This study was approved by the institutional review boards of National Yang-Ming University (approval number: YM108003E); Taipei Hospital, Ministry of Health and Welfare, Taiwan, R.O.C (approval number: TH-IRB-0019-0039) and National Yang-Ming University Hospital (approval number: 2019D04). Informed consent was obtained from all participants after they had received a thorough explanation of the study purpose, the procedures involved, and their rights as participants. Participation was completely voluntarily, and the recruited nurses were informed that they could withdraw at any time. In addition, they were assured that the confidentiality and anonymity of their responses would be maintained.

## 4. Results

### 4.1. Participant Characteristics

The vast majority of the participants were women (96.9%), and the average age was 32.44 ± 8.90 years. Approximately 64.6% of the nurses had a bachelor's degree, and > 50.5% were unmarried. The average amount of nursing experience was 9.90 ± 7.54 years, and of the nursing units that were considered, the most responses were obtained from nurses working in a critical care unit (20.7%; Table 1).

### 4.2. Results of Psychometric Tests

#### 4.2.1. Content Validity

The CAAS-C had excellent relevance and clarity, as indicated by S-CVI values of 0.99 and 0.94, respectively. The I-CVI value for each item was within the range 0.81–1.0, indicating that the final version of the CAAS-C had acceptable content validity.

#### 4.2.2. Construct Validity and Model Fit

**Figure 2:**
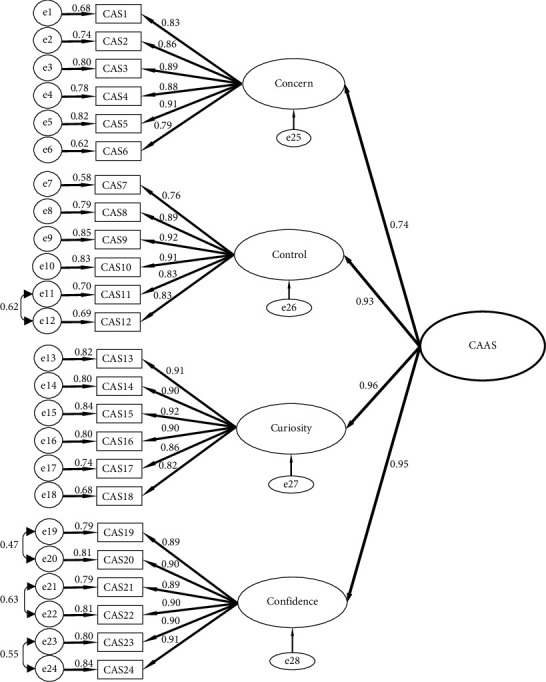
Confirmatory factor analysis of the Chinese version of the Career Adapt-Abilities Scale for clinical nurses.

CFA was performed to validate the scale constructs. The results of the initial estimation of the factor model revealed a poor fit (*χ*^2^ = 2660.84, *df* = 248, *p* < 0.001, *χ*^2^/*df* = 10.73, RMSEA = 0.129, GFI = 0.72, AGFI = 0.66, CFI = 0.87, and SRMR = 0.0603). Four error covariances were included; they were modifications determined through MIs [45]. The GFI parameters for the modified measurement model were as follows: *χ*^2^ = 608.86, *df* = 202, *p* < 0.001, *χ*^2^/*df* = 3.012, RMSEA = 0.059, GFI = 0.92, AGFI = 0.88, CFI = 0.98, and SRMR = 0.035. In the factor loading model, the first-order loadings for the items were within the range 0.76–0.92, indicating that each observed variable was well represented by its latent factor (Figure 2). Additionally, the second-order factor loadings for the latent variables were in the range 0.74–0.96, indicating that each latent variable was acceptable and was reflective of its construct.

#### 4.2.3. Internal Consistency Reliability

The internal consistency reliability of the CAAS-C was found to be excellent; the overall Cronbach's alpha coefficient was 0.96 (Table 2). The internal consistency reliability for the four subscales was also high, with Cronbach's alpha coefficients of 0.90 for concern, 0.94 for control, 0.95 for curiosity, and 0.96 for confidence (Table 2).

#### 4.2.4. Test–Retest Reliability

The 2-week test–retest reliability of the scale was assessed, and the results are presented in Table 3. The overall ICC for the CAAS-C was 0.82. The ICCs for the subscales for the test and retest were within the range 0.71–0.81. The ICCs were significant for the concern (*r* = 0.76; *p* < 0.001), control (*r* = 0.71; *p* < 0.001), curiosity (*r* = 0.72; *p* < 0.001), and confidence (*r* = 0.81; *p* < 0.001) subscales, indicating that the stability of the subscales ranged from acceptable to excellent (Table 3).

## 5. Discussion

Career adaptability is a key factor influencing nurses' career commitment and success as well as nurse retention. In this study, we translated the CAAS into traditional Chinese and validated the resultant CAAS-C. Our overall findings indicate that the translation and psychometric properties of the CAAS-C are appropriate for assessing career adaptability in a Taiwanese nurse population. We adopted a modified version of Brislin's guidelines [40] for scale translation, and we discovered that the CAAS-C has satisfactory content validity, test–retest reliability, and internal consistency [52].

The present study completed translation and validation in two phases. In the first phase, forward translation and back translation, we recruited qualified translators with bilingual skills, career-development-related knowledge, and experience with the CAAS. In the second phase, we consulted a panel comprising an expert familiar with the scale's constructs, a methodologist, and a translator to ensure semantic, conceptual and translation equivalence of the CAAS-C with the original scale [32, 40]. The results of the psychometric validation phase indicated that the CAAS-C has satisfactory reliability and validity. With respect to content validity, the panel of experts rated all items as relevant for assessing the career adaptability of Taiwanese nurses. The CAAS-C was determined to have acceptable content validity, indicating that the translated items accurately reflected the concepts of the original scale [53].

In descriptive analysis of the scale items, the lowest mean scores were obtained for the items “thinking about what my future will be like,” “planning how to achieve my goals,” and “preparing for the future.” This indicated that the nurse participants had relatively low interest in their current and future career and that nurses may not know what they want in their careers; therefore, they are often unable to self-regulate and unlikely to derive satisfaction from their career [26], which can lead to a high turnover rate [54]. Our findings indicate that nursing administrators and managers should design programs that can effectively inspire nurses and establish a supportive environment to increase the likelihood that nurses will have meaningful and interesting experiences in their work. This can in turn enhance their career interest and make them more likely to remain in the field of nursing.

According to the 2-week test–retest evaluation, the test–retest reliability of the four subscales ranged from acceptable to excellent, and the ICC for the overall scale was excellent [51]. These results regarding the reliability estimates are consistent with those of a previous study [37] and indicate that the CAAS-C exhibits substantial stability and reproducibility for assessing career adaptability in Taiwanese clinical nurses [55].

The Cronbach's alpha coefficients for the four subscales were all greater than 0.9, and that for the overall scale reached 0.96, indicating that the CAAS-C has excellent internal consistency [45]. This demonstrates that the scale items are strongly correlated with each other and is connected to the interrelatedness to the overall concept or construct [56]. Our results are consistent with those of other studies involving nurses, such as Pajic, et al. [24] and Sun, et al. [36]. Obtaining responses from a specific and homogeneous sample is likely to lead to a high Cronbach's alpha value and a high level of equivalence among items [57]. Therefore, to ensure high internal consistency, we determined whether removal of each item would result in a significant increase in the Cronbach's alpha coefficient for the overall scale; no significant increase was noted upon the removal of any of the items. This indicated that all items were relevant to the overall scale.

Regarding construct validity, we performed CFA to evaluate the latent structure of the constructs [58]. The first- and second-order career adaptability constructs had factor loading values of >0.50, which indicated satisfactory model fit. For the first-order items, most factor loading values, which indicate the extent to which observed variables (items) contribute to latent variables [45], were > 0.7. The highest factor loading values were obtained for items 9 and 15 (0.92). Item 9, “taking responsibility for my actions,” and item 15, “investigating options before making a choice,” had the strongest item-to-construct relationships. This finding, which is in line with that of Tian and Fan [59], indicates that individuals with a greater ability to adapt have a greater sense of autonomy over and curiosity toward their work.

Item 7, “keeping updated,” had the lowest factor loading (0.76). Other studies have reported similar findings [38, 59, 60]. This result indicates that the nurses did not believe that they had to continually update their job skills.

Regarding the second-order constructs, the results for the variance in the latent variables (factors) indicated that the loadings for all factors but control, confidence, and curiosity were higher than those for the factors on the international version of the CAAS, which were determined in validation studies conducted by Savickas and Porfeli [29] and Tien, et al. [38]. With the exception of those for the concern subscale, the factor loadings of the CAAS-C were similar to those of the international version of the CAAS and a version of the CAAS developed for Hungarian nurses (0.78) [24] but slightly lower than those of the CAAS developed for the general population of Taiwan (0.84) [38]. Generally, the scope of nursing practice is wide; consequently, nurses have numerous opportunities to select the specific field of health care they wish to pursue [61]. Therefore, nurses may not have concerns regarding their career development. This may have led to the concern subscale having a weak correlation with the constructs in this study. This result is similar to that of another validation study [24].

### 5.1. Strengths and Limitations

This study has several strengths. First, we ensured the psychometric accuracy of the CAAS-C through a two-phase translation and validation process; the conceptual equivalence of the scale was confirmed by five bilingual experts in the field of career development. Second, we assessed the test–retest reliability of the CAAS-C to ensure the reproducibility of its results and the stability of its constructs. Third, we assessed the construct validity of the CAAS-C through CFA of the second-order constructs. Finally, our sample was large, with the study including 584 clinical nurses in Taiwan.

This study also has several limitations. We performed purposive sampling in only two teaching hospitals in Taiwan. Therefore, the representativeness of the nursing sample may be limited. Future studies should employ random sampling and use a multicenter design to improve the generalizability of the results.

## 6. Conclusion

The present study substantially contributes to the literature by providing a traditional Chinese version of a psychometric tool for evaluating the career adaptability of Taiwanese nurses. Our findings indicate that the CAAS-C is a valid and reliable instrument that can effectively measure the career adaptability of clinical nurses in Taiwan. The CAAS-C and its four subscales have excellent test–retest reliability and internal consistency reliability. Moreover, the CAAS-C has satisfactory content and constructs validity. Therefore, the scale can be used to evaluate Taiwanese nurses' career adaptability. Future studies should conduct psychometric evaluations to validate the applicability of the scale for other health-care professionals and contexts to expand its use and enable comparisons of different vocational groups.

## 7. Implications for Nursing Management

The CAAS-C can help Taiwanese nursing managers measure the career adaptability of nurses. In addition, it can guide them in designing training and counseling programs to improve nurses' career adaptability to ensure they have sufficient personal resources and remain in the nursing workforce. The scale can also be used to assess the effectiveness of career adaptability resources in various health-care settings to provide nursing administrators with crucial information that can guide the development and implementation of strategies for promoting adaptability in nurses. Hospital nurse administrators and managers can employ this tool to understand nurses' adaptive behavior and capacity and use this information to strategically strengthen their adaptive capacity; in addition, they can use the information to establish a supportive and inspiring work environment, which can lead to nurses perceiving their work to be meaningful and can thereby reduce turnover. Furthermore, this qualitative tool can be used to improve the understanding of career adaptability in the nursing context.

## Figures and Tables

**Table 1:** Participant characteristics (*N* = 584).

Variables	*n*	%	M (SD)	Range
Gender				
Male	18	3.1		
Female	566	96.9		
Age			32.44 (8.90)	21.0–60.0
21–25 years	145	24.8		
26–30 years	137	23.5		
31–35 years	89	15.2		
36–40 years	114	19.5		
41–45 years	57	9.8		
Above 46 years	42	7.2		
Educational level				
Associate degree	195	33.4		
Bachelor degree	377	64.6		
Graduate degree	12	2.1		
Marital status				
Unmarried	295	50.5		
Married	220	37.7		
Unanswered	49	8.4		
Divorce/widowed	20	3.4		
Years of nursing			9.90 (7.54)	0.08–34.75
0-1	24	4.1		
1–5	207	35.4		
6–10	129	22.1		
11–15	96	16.4		
16–20	79	13.5		
21^+^	49	8.4		
Work of units				
ICU/RCC	121	20.7		
Medical	84	14.4		
Surgical	62	10.6		
OR	59	10.1		
OPD	51	8.7		
ER	54	9.2		
HDR	32	5.5		
PSY	26	4.5		
PED/GYN	56	9.6		
Community health	28	4.8		
Hospice	7	1.2		
Nursing home	4	0.7		

Abbreviations: M, mean; SD, standard deviation.

**Table 2:** CAAS-C: Descriptive statistics and internal consistency (*N* = 548).

Construct/item	M	SD	Corrected item-total correlation	Cronbach's *α* if item is deleted	Cronbach's *α*
Concern	2.90	0.83			0.90
1. Thinking about what my future will be like	2.77	0.95	0.74	0.88	
2. Realizing that today's choices shape my future	2.92	0.95	0.70	0.89	
3. Preparing for the future	2.87	0.91	0.73	0.89	
4. Becoming aware of the educational and career choice that I must make	2.92	0.93	0.81	0.87	
5. Planning how to achieve my goals	2.85	0.94	0.73	0.88	
6. Concerned about my career	3.05	0.94	0.69	0.89	
Control	3.30	0.77			0.94
7. Keeping update	2.99	0.88	0.71	0.95	
8. Making decision by myself	3.22	0.87	0.86	0.93	
9. Taking responsibility for my action	3.38	0.88	0.88	0.93	
10. Sticking up for my beliefs	3.26	0.85	0.87	0.93	
11. Counting on myself	3.46	0.90	0.83	0.93	
12. Doing what's right for me	3.49	0.88	0.82	0.93	
Curiosity	3.35	0.79			0.95
13. Exploring my surroundings	3.40	0.88	0.87	0.95	
14. Looking for opportunities to grow as a person	3.28	0.88	0.86	0.95	
15. Investigating options before making a choice	3.40	0.88	0.89	0.94	
16. Observing different ways of doing things	3.45	0.89	0.88	0.94	
17. Probing deeply into questions I have	3.28	0.85	0.85	0.95	
18. Becoming curious about new opportunities	3.29	0.89	0.81	0.95	
Confidence	3.36	0.79			0.96
19. Performing tasks efficiently	3.37	0.87	0.87	0.96	
20. Taking care to do thing well	3.45	0.87	0.89	0.96	
21. Learning new skills	3.35	0.86	0.89	0.96	
22. Working up to my ability	3.34	0.86	0.89	0.96	
23. Overcoming obstacles	3.32	0.84	0.89	0.96	
24. Solving problems	3.34	0.83	0.91	0.96	

**Table 3:** Test–retest correlation for the Chinese version of the Career Adapt-Abilities Scale (*n* = 37).

Variables	Pre-test		Post-test		ICC	*p*
	Mean	SD	Mean	SD		
Career Adapt-Abilities Scale	3.27	0.66	3.19	0.55	0.82	< 0.001⁣^∗∗∗^
Concern	2.94	0.65	2.89	0.65	0.76	< 0.001⁣^∗∗∗^
Control	3.40	0.76	3.23	0.53	0.71	< 0.001⁣^∗∗∗^
Curiosity	3.38	0.79	3.24	0.62	0.72	< 0.001⁣^∗∗∗^
Confidence	3.38	0.71	3.39	0.61	0.81	< 0.001⁣^∗∗∗^

Abbreviation: ICC, intraclass correlation coefficient.

⁣^∗∗∗^*p* < 0.001.

## Data Availability

The data sets used and analyzed in the present study are available from the corresponding author upon reasonable request.
